# CRF transcription factors in the trade-off between abiotic stress response and plant developmental processes

**DOI:** 10.3389/fgene.2024.1377204

**Published:** 2024-04-16

**Authors:** Davide Gentile, Giovanna Serino, Giovanna Frugis

**Affiliations:** ^1^ Institute of Agricultural Biology and Biotechnology (IBBA), National Research Council (CNR), Rome, Italy; ^2^ Department of Biology and Biotechnology ‘Charles Darwin’, Sapienza University of Rome, Rome, Italy

**Keywords:** CRF transcription factors, abiotic stress response, oxidative stress, development, cytokinin, auxin, senescence, photosynthesis

## Abstract

Climate change-induced environmental stress significantly affects crop yield and quality. In response to environmental stressors, plants use defence mechanisms and growth suppression, creating a resource trade-off between the stress response and development. Although stress-responsive genes have been widely engineered to enhance crop stress tolerance, there is still limited understanding of the interplay between stress signalling and plant growth, a research topic that can provide promising targets for crop genetic improvement. This review focuses on Cytokinin Response Factors (CRFs) transcription factor’s role in the balance between abiotic stress adaptation and sustained growth. CRFs, known for their involvement in cytokinin signalling and abiotic stress responses, emerge as potential targets for delaying senescence and mitigating yield penalties under abiotic stress conditions. Understanding the molecular mechanisms regulated by CRFs paves the way for decoupling stress responses from growth inhibition, thus allowing the development of crops that can adapt to abiotic stress without compromising development. This review highlights the importance of unravelling CRF-mediated pathways to address the growing need for resilient crops in the face of evolving climatic conditions.

## 1 Introduction

Climate change is leading to increased variability and extremes in environmental conditions, which can trigger abiotic stress responses in plants (Bibi and Rahman, 2023; Eckardt et al., 2023). Environmental stressors such as low or high temperature, drought or flooding, and nutrient deficiency can be hostile to plant growth and development, leading to a significant reduction in crop yield and quality (He et al., 2018; Rai et al., 2021).

Defense against stress and active growth suppression are two complementary strategies by which plants respond to adverse environments. When exposed to unfavourable conditions plants activate stress response pathways and, depending on the plant developmental stage, accelerate senescence at the expense of plant growth. Although beneficial for plant survival, active growth inhibition is often undesirable for crop productivity. This stress-development trade-off is therefore of significant importance in agriculture to select more resilient crops that maintain their productive characteristics in increasingly variable and extreme environmental conditions. Stress-responsive genes, most importantly hormonal signalling genes and transcription factors (TFs), play a crucial role in how plants respond to environmental stressors (Ahanger et al., 2017; He et al., 2018). Major efforts in precision breeding and genome editing have been employed to target these genes to develop crops with greater resilience to environmental stress (Sinha et al., 2021; Nerkar et al., 2022). However, compared with the knowledge on how plants defend against abiotic stress, more limited information is available on how stress signalling regulates plant growth and *vice versa*. The regulatory networks for stress response and growth regulation crosstalk act at multiple levels, some of them well characterized such as those involving abscisic acid (ABA), while others remain elusive. Understanding and manipulating the regulatory networks that control growth-defense trade-off could pave the way for uncoupling stress response and growth, thus making it possible to enhance stress resistance without compromising crop productivity (Zhang et al., 2020).

In this review, we will discuss the possible role of Cytokinin Response Factors (CRFs) as key players in the trade-off that takes place between stress response and plant growth, particularly in the delicate equilibrium between photosynthesis and the redox status, chlorophyll maintenance and senescence.

CRFs are a small family of transcription factors (Rashotte et al., 2006), that is present ubiquitously in land plants as part of the larger AP2/ERF (APETALA2/ethylene-responsive element binding factors) TFs family. TFs represent the primary regulatory level in determining an organism’s physiological response to environmental stimuli, as TFs expression is modulated by endogenous and exogenous factors, and in turn regulates the expression of downstream target genes, enabling contextual flexibility and response specificity (Baillo et al., 2019). AP2/ERF TFs, together with WRKY, MYB, NAC, and bZIP families, have been implicated in abiotic stress responses, and loss or gain of function alleles of genes from those families are often associated with enhanced tolerance in both model and crop plants (Wang et al., 2016). AP2/ERF are plant-specific TFs and are known to regulate diverse processes of environmental stress responses, such as cold, heat, drought, salinity, and osmotic stress. Furthermore, numerous studies have documented that genetically modified plants overexpressing AP2/ERF family TFs have improved tolerance to abiotic stresses (Zhu, 2002; Feng et al., 2020).

CRFs consist of one AP2/ERF DNA binding domain, a strongly conserved CRF domain involved in protein-protein interaction that is unique to CRFs, a putative mitogen-activated protein kinase (MAPK) phosphorylation motif, and a variable C-terminal region. CRFs have become increasingly recognized as key TFs in responding to abiotic stresses in many plant species (Hallmark and Rashotte, 2019). Furthermore, several members of the CRF family from various plant species are induced by oxidative stress (OS), which is considered one of the most crucial consequences of abiotic stress (Hasanuzzaman et al., 2020; Maurya, 2020). CRFs are also subsidiary elements of the cytokinin (CK) signalling pathway. CKs are plant hormones that regulate several important aspects of plant development and growth such as cell division and differentiation, shoot development and organogenesis, sink/source relationship, and nutrient uptake (Bailey-Serres and Mittler, 2006; Kieber and Schaller, 2014; del Río, 2015; Li et al., 2021). Growing evidence also supports a role of CKs in abiotic stress response and in regulating plant-microbe interactions (Cortleven et al., 2019). Overall, these findings point to CKs and CRF TFs as possible key players in the trade-off between growth and stress response.

## 2 CRFs in hormonal signalling

Cytokinin levels in plants are regulated by biosynthesis and inactivation pathways. CKs are perceived by membrane-localized histidine kinase receptors (CHKs). This signal is transferred through a His-Asp phosphorelay involving histidine phosphotransfer proteins (HPTs), to activate a family of transcription factors, the cytokinin Response Regulators (RRs), in the nucleus (Kieber and Schaller, 2014). Type-A RRs act as negative regulators of CKs signalling whereas type-B RRs are positive regulators in this pathway. CRFs interact functionally with the CKs pathway (Rashotte et al., 2006). All CRFs link to the CKs response through their distinctive CRF domain, which enables CRFs proteins to directly interact with most HPTs (AHP1-5), and with specific type-B RRs in *Arabidopsis* (Cutcliffe et al., 2011). This interaction likely allows CRFs to modulate the activity of CKs signalling pathway components, fine-tuning downstream cytokinin-responsive gene expression and physiological responses linked to CK. CRFs can also form homodimers, and heterodimerize with each other in any combination, as shown in *Arabidopsis* (Cutcliffe et al., 2011) and *Solanum lycopersicum* (Shi et al., 2012), adding a further degree of complexity and flexibility to the CKs signalling pathway.

Although initially identified as AP2 TFs that are transcriptionally upregulated by CKs, only a subset of CRFs respond to CKs. CK inducibility was observed for *AtCRF1, AtCRF2, AtCRF5, and AtCRF6* in *Arabidopsis* (Rashotte et al., 2006; Zwack et al., 2012), *SlCRF1, SlCRF2, SlCRF3* and *SlCRF5* in *Solanum lycopersicum* (Shi et al., 2012), most of CRFs from *Brassica rapa* (Liu et al., 2013) and *McCRF1* in *Marshallia caespitosa,* (Melton et al., 2019). Importantly, the induction of *AtCRF2* and *AtCRF5* by CK was shown to be dependent on the type-B RRs in *Arabidopsis*, as those genes are not induced in the type-B mutant *arr1,12* (Rashotte et al., 2006)*.* Moreover, RNA-seq analysis showed that genes differentially regulated in *crf1,3,5,6* mutant roots are highly enriched for CKs-regulated genes (Raines et al., 2016). These findings indicate that CRF TFs and CKs signalling are intimately interconnected, as CRFs are both downstream and upstream of the CKs transcriptional cascade, and physically interact with components of the CKs signal transduction at protein level.

CKs extensively interact with other plant hormones, leading to complex crosstalk networks that regulate various aspects of plant development (El-Showk et al., 2013). The nature and extent of these interactions can differ across plant species. CKs also influence cell-to-cell auxin (IAA) transport by modification of the expression of several IAA transport components and thus to modulate IAA distribution during root development in *Arabidopsis* (Dello Ioio et al., 2008; Pernisová et al., 2009; Ruzicka et al., 2009; Marhavý et al., 2011).

*Arabidopsis* CRFs also participate in the regulation of auxin transport directly regulating the expression of PIN-formed (PIN) auxin efflux carrier proteins. Specifically, gene expression analyses of loss-of-function mutants of AtCRF2, AtCRF3, and AtCRF6 indicate that AtCRF2 and AtCRF6 are positive regulators of PIN1 and PIN7, while AtCRF3 is a negative regulator of those genes (Šimášková et al., 2015). Indeed, analyses of single, double or triple *crf1, crf2, crf5,* and *crf6* mutants revealed abnormal leaf vascular patterning (Zwack et al., 2012), increased occurrence of double embryos and reduced root meristem size*,* similar to the defects observed in auxin transport and signalling mutants (Šimášková et al., 2015). AtCRF2, AtCRF3, and AtCRF6 are also required to regulate *PIN1* expression during inflorescence development, and this regulation is necessary for pistil elongation and ovule number (Cucinotta et al., 2016). Moreover, during shoot formation and roots embryogenesis, AtCRF2 acts genetically downstream of MONOPTEROS/ARF5 (Auxin Response Factor 5), a transcription factor that mediates auxin-responsive gene expression and promotes auxin transport (Subbiah and Reddy, 2010; Ckurshumova et al., 2014). This evidence further indicates that CRFs play a key role in the auxin-CKs crosstalk.

Interaction between CRFs and PIN-formed (PIN) proteins has not been studied in species other than *Arabidopsis*. However, the CRFs function in auxin regulation might be conserved since in soybean GmCRF4a is both required for the expression of several auxin biosynthetic YUCCA genes (*GmYUC4a, GmYUC4b, GmYUC10a*), and for repression of the negative regulator of auxin signalling *GmIAA14a* (Xu et al., 2022).

## 3 CRFs in the trade-off between stress and plant growth

Members of the CRF family from *Arabidopsis* (Zwack et al., 2013; Zwack et al., 2016b; Inupakutika et al., 2016; Hieno et al., 2019; Hughes et al., 2021), tomato (Gupta and Rashotte, 2014; Shi et al., 2014; Hughes et al., 2021), *M. caespitosa* in the Asteraceae (Melton et al., 2019), and *Tamarix hispida* in Tamaricaceae (Qin et al., 2017), are modulated by oxidative stress. Oxidative stress is a common outcome of various abiotic stresses (Huang et al., 2019). One of the most crucial consequences of abiotic stress is the disturbance of the equilibrium between the generation of Reactive Oxygen Species (ROS) and antioxidant defence systems (Maurya, 2020), which lead to the excessive production and accumulation of ROS. The resulting OS stress damage can disrupt normal cellular processes and alter cell structure, leading to changes in plant architecture and morphogenesis, affecting crops yield and organoleptic properties (Sahu et al., 2022).

In *Arabidopsis AtCRF2*, *AtCRF5, AtCRF6, AtCRF7,* and *AtCRF8* are induced by OS (Table 1). *AtCRF6* transcription is activated by several OS-inducing treatments including methyl viologen, UV-B light, antimycin-A, and H_2_O_2_ (Inzé et al., 2012; Zwack et al., 2013; Zwack et al., 2016b). Induction of *AtCRF6,* and its paralog *AtCRF5,* has also been associated with Mitochondrial Retrograde Signalling (MRS), a signalling cascade that occurs upon mitochondrial function disturbance by stress. This transcriptional activation is dependent on ANAC017, a membrane-bound TF that relocalizes to the nucleus in response to the antimycin-A treatment and physically binds the promoters of *AtCRF5* and *AtCRF6* to activate their transcription (Ng et al., 2013).

**TABLE 1:** CRFs from various species are involved in both stress responses and developmental programs and can be responsive to CKs and OS or both.

Genes	Species	CKs/OS induction	Stress response	Developmental response	References
*AtCRF1*	*Arabidopsis thaliana*	CKs	Salt	Root Development, Shoot Growth Inhibition, Senescence Promotion	Keshishian (2018), Raines et al. (2016), Zwack et al. (2012)
*AtCRF2*	*Arabidopsis thaliana*	CKs, OS	Pathogen, Cold, Oxidative, Salt	Chloroplast Division, Root and Reproductive Development, Senescence Promotion	Rashotte et al. (2006), Okazaki et al. (2009), Šimášková et al. (2015), Cucinotta et al. (2016), Inupakutika et al. (2016), Jeon et al., 2016; Kwon (2016), Keshishian et al. (2022)
*AtCRF3*	*Arabidopsis thaliana*		Cold	Root and Reproductive Development, Shoot Growth Inhibition, Senescence Promotion	Šimášková et al. (2015), Cucinotta et al. (2016), Jeon et al. (2016), Raines et al. (2016)
*AtCRF4*	*Arabidopsis thaliana*		Cold	Nitrogen Signalling	Zwack et al. (2016a), Brooks et al. (2019)
*AtCRF5*	*Arabidopsis thaliana*	CKs, OS	Pathogens, Oxidative	Root Development, Shoot Growth Inhibition, Senescence Promotion	Rashotte et al. (2006), Liang et al. (2010), Raines et al. (2016), Hughes et al. (2021)
*AtCRF6*	*Arabidopsis thaliana*	CKs, OS	High Light, Osmotic, UV-B, Cold, Oxidative, Dark, Drought, Heath, Oxidative	Root and Reproductive Development, Senescence Inhibition, Potassium Transport	Rashotte et al. (2006), Winter et al. (2007), Zwack et al. (2013), Šimášková et al. (2015), Cucinotta et al. (2016), Hughes et al. (2020)
*AtCRF7*	*Arabidopsis thaliana*	OS	Oxidative		Hieno et al. (2019)
*AtCRF8*	*Arabidopsis thaliana*	OS	Phosphate Starvation, Oxidative		Ramaiah et al. (2014), Hieno et al. (2019)
*AtCRF9*	*Arabidopsis thaliana*			Reproductive Development, Chlorophyll Retention	Swinka et al. (2023)
*SlCRF1*	*Solanum lycopersicum*	CKs	Pathogen, Salt, Cold, Heat, Flood, Drought		Gu et al. (2002), Shi et al. (2012), Shi et al. (2014)
*SlCRF2*	*Solanum lycopersicum*	CKs, OS	Flood, Drought, Oxidative		Shi et al. (2012), Shi et al. (2014)
*SlCRF3*	*Solanum lycopersicum*	CKs, OS	Drought, Oxidative, Cold		Shi et al. (2012), Shi et al. (2014)
*SlCRF4*	*Solanum lycopersicum*		Salt		Shi et al. (2012)
*SlCRF5*	*Solanum lycopersicum*	CKs, OS	Flood, Drougt, Cold, Oxidative		Shi et al. (2012), Shi et al. (2014), Hughes et al. (2021)
*SlCRF6*	*Solanum lycopersicum*	CKs	Salt		Shi et al. (2012)
*SlCRF7, SlCRF8, SlCRF9*	*Solanum lycopersicum*	CKs			Shi et al. (2012)
*ThERF1*	*Tamarix hispida*		Salt, Drought, Oxidative		Wang et al. (2014), Qin et al. (2017)
*TSI1*	*Nicotiana tabacum*		Pathogen, Osmotic		Park et al. (2001)
*BrCRF1, BrCRF19*	*Brassica rapa*		Drought		Kong et al. (2018)
*BrCRF2*	*Brassica rapa*	CKs	Drought		Kong et al. (2018)
*BrCRF5, BrCRF21*	*Brassica rapa*		Salt		Kong et al. (2018)
*BrCRF7, 10–15*	*Brassica rapa*	CKs			Kong et al. (2018)
*BnaCRF8s*	*Brassica napus*		Phosphate Starvation	Root Development	Wang et al. (2020)
*CaPOS1*	*Capsicum annum*			Fruit Size (Cell Expansion), Flower Size, Seed Development	Wang et al. (2022)
*GmCRF4, GmCRF12, GmCRF21*	*Glycine max*		Salt		Duan et al. (2023)
*GmCRF2, GmCRF3, GmCRF5*	*Glycine max*		Cold		Duan et al. (2023)
*GmCRF20*	*Glycine max*			Plant Height	Duan et al. (2023)
*GmCRF6, GmCRF8*	*Glycine max*		Drought		Duan et al. (2023)
*McCRF1*	*Marshallia caespitosa*	CKs, OS	Oxidative		Melton et al. (2019)
*POS1*	*Physalis floridana, P. philadelphica*			Flower Size, Fruit Size (Cell Expansion)	Wang et al. (2014), Wang et al. (2022)
*PtERF85*	*Populus tremula x tremuloides*			Xylem Expansion, Secondary Cell Wall Deposition	Seyfferth et al. (2021)
*QsCRF3*	*Quercus suber*			Embryo Development	Capote et al. (2019)

Increased OS tolerance of the *arr6*, *arr9*, *arr11*, *log7*, and *abcg14* loss-of-function mutants are similar to those of plants overexpressing *AtCRF6,* showing a lesser reduction of photosystem II efficiency and in chlorophyll content compared to the WT upon OS induction, whereas *crf6* loss-of-function plants have an opposite OS-response phenotype. Differentially expressed genes in either *crf5* and *crf6 Arabidopsis* mutants, or tomato SlCRF5-antisense knockdown, are highly enriched in CKs-related genes. It was therefore proposed that *AtCRF6* and *AtCRF5* mediate the response to OS, partly through the repression of a set of genes involved in cytokinin metabolism (*LOG7*), transport (*ABCG14*), and signalling (*ARR6*, *ARR9*, *ARR11*) by AtCRF6, and cytokinin glucosylation (*UGT76C2*) by AtCRF5 to attenuate cytokinin signalling as part of an adaptive stress response (Zwack et al., 2016b; Hughes et al., 2021). AtCRF6, AtCRF5, and SlCRF5 were also shown to regulate CKs levels upon OS induction (Hughes et al., 2021), in addition to the CK response regulation layer provided by protein-protein interactions with AHP and RRs. Importantly, an interaction between AtCRF6 and the promoter of *ARR6* was identified, suggesting that AtCRF6 may directly regulate the transcriptional activity of some of its CKs-related targets (Zwack et al., 2016b).

Amongst the 12 CRFs identified in *Arabidopsis, AtCRF2* and At*CRF8* were also reported as redox-response transcription factors (Inupakutika et al., 2016), and *AtCRF7* was found amongst the hydrogen peroxide (H_2_O_2_)-responsive TFs identified by microarray analysis (Hieno et al., 2019). Although no further studies investigated transcriptional response to oxidative stress for the other members of the CRF family, most of them are regulated during various abiotic responses (Table 1), within which they could be induced by OS as a secondary messenger for abiotic stress response.

Interestingly, the *Tamarix hispida ThCRF1* is induced upon salt stress and transcriptionally activates genes involved in the biosynthesis of proline and trehalose, and in ROS scavenging (superoxide dismutase, SOD; peroxidase, PRX), which lead to enhanced osmoprotectants content and antioxidant defence (Wang et al., 2014; Qin et al., 2017). This might indicate that CRFs are induced by OS and that in turn they transcriptionally activate genes of the ROS scavenging pathway to counteract stress-induced cellular damage.

## 4 CRFs as target genes to delay senescence and reduce yield penalty under abiotic stress

Abiotic stresses accelerate leaf senescence, thus resulting in reduced photosynthetic efficiency, crop yield and quality (Tan et al., 2023). CKs have long been known to inhibit leaf senescence (Richmond and Lang, 1957; Gan and Amasino, 1995) in model and crop species (Ori et al., 1999; McCabe et al., 2001). In tobacco, the expression of the CK biosynthetic enzyme isopentenyltransferase (IPT) driven by stress and maturation-inducible promoter enhances drought tolerance by delaying leaf senescence (Rivero et al., 2007). Senescent cells are characterized by increased ROS production and chlorophyll (Chl) degradation rate. ROS can cause DNA damage and activate Senescence-Associated Genes (SAGs) (Tan et al., 2023), while chlorophyll degradation allows plants to remobilize nitrogen (Khanna-Chopra, 2012).

AtCRF6 was shown to play a role in delaying leaf senescence (Zwack et al., 2013). Under senescence conditions, leaves overexpressing *AtCRF6* retain more Chl than those of the WT. Expression analyses indicate that *AtCRF6* is highly expressed in the veins of mature leaves and that this expression decreases with age (Zwack et al., 2013) as seen for other senescence-related genes (Miryeganeh, 2022). While AtCRF6 is a negative regulator of leaf senescence, AtCRF1, AtCRF3, and AtCRF5 act as positive regulators, since lines overexpressing these genes display early leaf senescence (Raines et al., 2016). The *crf1,3,5,6* multiple knock-out line exhibits delayed senescence respect to the WT when leaf yellowing was compared (Raines et al., 2016), whereas AtCRF2-OX plants, besides showing accelerated senescence in rosette leaves, displayed enhanced age-dependent cell death and increased expression of the senescence-associated genes *SAG12* and *SAG113* (Kwon, 2016). However, in a previous study, CRF2 overexpression lines, as well as CK-treated plants, were shown to have accelerated chloroplast division rate (Okazaki et al., 2009), that is a trait associated with enhanced photosynthetic activity (Vercruyssen et al., 2015). The contribution of the single CRFs to these phenotypes is still unclear and may depend on complex protein-protein interactions. However, all these studies indicate that several members of the CRF family may affect CK signalling in the equilibrium between active photosynthesis maintenance and senescence in different plant tissues. Consistently, the overexpression of another *Arabidopsis* CRF, *AtCRF9*, involved in reproductive development, promotes chlorophyll retention in dark-induced senescence assays (Swinka et al., 2023). AtCRF9 was shown to act as a transcriptional repressor of the cytokinin primary response gene *ARR6,* similarly to AtCRF6 (Zwack et al., 2016b). Searching for genotypes that display enhanced expression of those CRFs promoting chlorophyll retention in crops may allow the identification of valuable allelic variants for breeding and genome editing strategies.

The balance between the induction of leaf senescence and the maintenance of photosynthesis can play a major role in drought tolerance and in preserving crop yields during stress in both monocot and dicot crop species (Kamal et al., 2019; Baldoni et al., 2021; Tan et al., 2023). In cereals, the stay-green response (SGR) is a secondary trait that enables crop plants to maintain their green leaves and photosynthesis capacity for a longer time after anthesis, especially under drought and heat stress conditions. Several mutants displaying the stay-green trait derive from the inactivation of genes involved in chlorophyll breakdown (Kamal et al., 2019).

A recent study identified the two principal antagonistic transcriptional networks that control photosynthesis in the leaves of *Cichorium endivia* (Testone et al., 2019). The main photosynthesis-driven TF regulatory network involves light signal transduction to promote the expression of photosynthesis master regulators and downstream genes. The other relates to photooxidative stress, chloroplast-nucleus retrograde signalling (RS), unfolded protein response (UPR) (Chan et al., 2016) and senescence. This is consistent with the large increase in the production of ROS derived from light-driven energy transfer and electron transport during the photosynthetic process (Foyer, 2018). Several developmental genes, including hormone response genes, were found to associate with either the photosynthesis-promoting cluster or the oxidative stress module (Testone et al., 2019). This points to a tight connection between plant development and the maintenance of the equilibrium between photosynthesis and oxidative stress/senescence, and to a major role of hormone signalling in integrating these antagonistic transcriptional responses. Interestingly, amongst the major hubs associated with the photosynthetic function, there were genes homologous to type-B RR promoting CK signalling, including *ARR12*, several ARFs and *CRF2*. A homolog of *MONOPTEROS/ARF5* was instead associated to the oxidative stress transcriptional module (Testone et al., 2019). These findings may indicate that the regulatory circuit involving specific auxin-cytokinin response regulators, and their associated CRFs, could play a central role in the fine equilibrium between photosynthesis maintenance and oxidative stress/senescence in both model and crop species.

## 5 Concluding remarks

**FIGURE 1:**
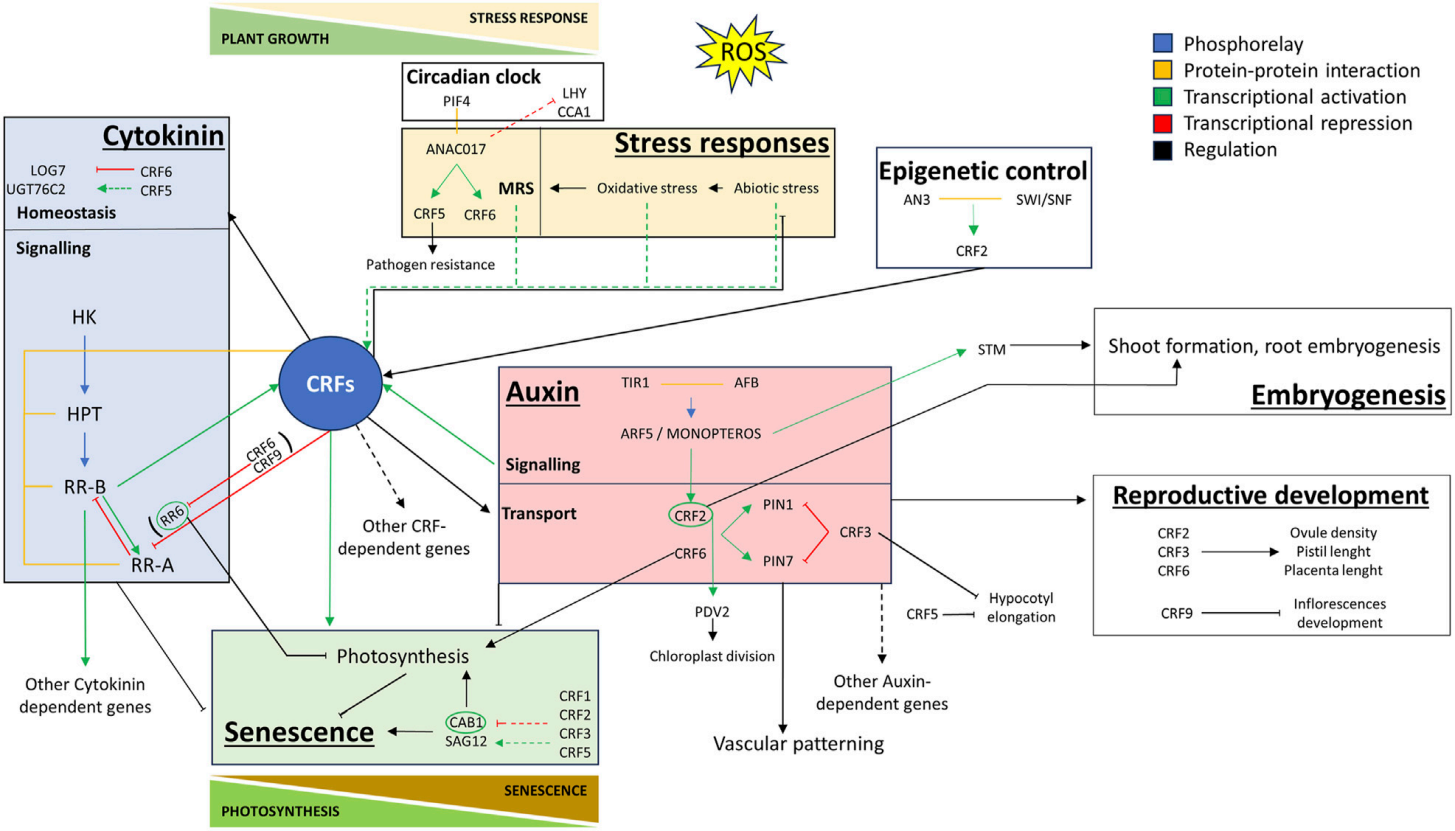
Graphical model illustrating the complex roles of Cytokinin Response Factors (CRFs) in the trade-off between abiotic stress response and plant developmental processes. Coloured boxes highlight the role of specific CRFs in stress responses (yellow box), CK/IAA hormonal crosstalk (light blue and pink boxes) and senescence (green box). The blue box focuses on the involvement of CRFs in cytokinin homeostasis and signalling. The pink box depicts the regulation of auxin transport exerted by CRFs. Lastly, the green box emphasizes the trade-offs between photosynthesis and senescence regulated by CRFs. This model provides a comprehensive view of the multifaceted roles of CRFs in integrating endogenous and exogenous signals to regulate plant development. YELLOW BOX: Under various stress conditions, specific CRFs are transcriptionally regulated and have a role in regulating the specific stress response (Table 1). OS is a common outcome of various abiotic stress responses. During OS response, ANAC017 is released from the mitochondrial membrane and binds to the promoters of CRF5 and CRF6, activating their transcription. ANAC017 also interacts with PIF4 at the protein level, showing a diurnal expression pattern, and repressing circadian regulators LHY and CCA1. CRF5 is also involved in pathogen resistance. LIGHT BLUE BOX: CKs are perceived by membrane-localized histidine kinase receptors (CHKs) and are transduced through histidine phosphotransferase proteins (HPTs) to activate response regulators (RRs) in the nucleus (Kieber and Schaller, 2014). Type-A RRs act as negative regulators of CKs signalling whereas type-B RRs act as transcription factors and are positive regulators in this pathway. CRF5 represses UGT76C2, responsible for cytokinin N-glucosylation, and CRF6 represses genes responsible for CK signalling (ARR6, ARR9, ARR11), biosynthesis (LOG7), and transport (ABCG14). The CRFs exhibit protein-level interactions with HPT, among themselves in various combinations, and with specific RRs, providing contextual flexibility and response specificity to the CK signalling pathway. CRFs 2, 5, and 6 are transcriptionally activated by CKs in a manner dependent on AHK receptors and specific RRs (ARR1 and ARR12). PINK BOX: AtCRF2 and AtCRF6 positively regulate PIN1 and PIN7, while AtCRF3 acts as a negative regulator. AtCRF2 is also auxin-responsive, downstream of MONOPTEROS/ARF5, and participates in root embryogenesis and shoot formation together with SHOOT MERISTEMLESS (STM). CRF2, 3 and 6 are positive regulators of ovule density, pistil length, and placenta length. CRF9 is a negative regulator of silique and seed development and shoot apical meristem floral transition. CRFs are involved also in other auxin-regulated processes such as hypocotyl elongation and vascular patterning. GREEN BOX: CRF2 is under the epigenetic control of SWI/SNF chromatin remodelling complex. CRF2 is a positive senescence regulator, PDV2 is downstream of CRF2 and promotes chloroplast division. CRF6 positively regulates photosynthetic activity and delays senescence by repressing ARR6, a negative regulator of chlorophyll retention. Conversely, crf1/3/5 knockout lines show reduced expression of the senescence regulator SAG12, increased expression of the photosynthesis regulator CAB1, and an early onset of senescence.

Functional studies of the CRF TFs, mainly conducted in *Arabidopsis* and to a limited extent in tomato, point to a key CRFs role in modulating CK-IAA hormonal crosstalk during both development and abiotic stress response (Figure 1). CRFs regulate cytokinin signalling through protein-protein interaction with HPTs and RRs, and at transcriptional level downstream of type-B and upstream of type-A response regulators (light blue box in Figure 1). Also, some CRFs act downstream of auxin response factors and in turn regulate auxin transport through PINs (pink box in Figure 1). CRFs function and expression also respond to oxidative stress and redox status (yellow box in Figure 1) and may connect developmental and abiotic stress responses mediated by ROS. The CRFs role in the delicate balance between photosynthesis maintenance and the onset of senescence (green box in Figure 1) is of particular importance in the possibility of obtaining crop species resilient to adverse environmental conditions without yield penalty. This CRFs role may be exerted through the transcriptional regulation of type-A response regulators in the CK pathway, particularly *ARR6* in *Arabidopsis*. Type-A response regulators are rapidly induced by CKs and mediate a feedback mechanism by which the plant decreases its sensitivity to the hormone (To et al., 2004). Mutants in type-A response regulators display delayed senescence. The ability of some CRFs to up- or downregulate *ARR6* may subtend a key role of CRFs in modulating cell sensitivity to CKs by regulating the amplitude and duration of the signal. This could represent a key regulatory step affecting both developmental processes and abiotic stress responses. The characterization of CRFs in crops, mostly neglected so far, with the identification of CRFs allelic variants in either protein functional domains (CRF protein-protein interaction domain, AP2 DNA binding domain, C-terminus transactivation domain) or important regulatory domains (ANAC017 binding site, CK- and redox-responsive elements), may open new perspectives in the genetic improvement of crop resilience traits based on a candidate gene approach. In the genomics area, many genetic diversity resources are available for most cultivated species, both wild relatives and domesticated cultivars, which could be exploited to this purpose.
